# Prospectively ECG-Triggered Sequential Dual-Source Coronary CT Angiography in Patients with Atrial Fibrillation: Influence of Heart Rate on Image Quality and Evaluation of Diagnostic Accuracy

**DOI:** 10.1371/journal.pone.0134194

**Published:** 2015-07-29

**Authors:** Lin Yang, Lei Xu, U. Joseph Schoepf, Julian L. Wichmann, Mary A. Fox, Jing Yan, Zhanming Fan, Zhaoqi Zhang

**Affiliations:** 1 Department of Radiology, Beijing Anzhen Hospital, Capital Medical University, Beijing, China; 2 Department of Radiology and Radiological Science, Medical University of South Carolina, Charleston, SC, United States of America; 3 Siemens Healthcare China, 278 Zhouzhu Road, Shanghai, China; Shenzhen institutes of advanced technology, CHINA

## Abstract

**Objectives:**

To evaluate the effects of mean heart rate (HR) and heart rate variation (HRV) on image quality and diagnostic accuracy of prospectively ECG-triggered sequential dual-source coronary CT angiography (CCTA) in patients with atrial fibrillation (AF).

**Methods:**

Eighty-five patients (49 women, 36 men; mean age 62.1±9.5 years) with persistent AF underwent prospectively ECG-triggered sequential second-generation dual-source CCTA. Tube current and voltage were adjusted according to body mass index (BMI) and iterative reconstruction was used. Image quality of coronary segments (four-point scale) and presence of significant stenosis (>50%) were evaluated. Diagnostic accuracy was analyzed in 30 of the 85 patients who underwent additional invasive coronary angiography (ICA).

**Results:**

Only 8 of 1102 (0.7%) segments demonstrated poor image quality. No significant impact on image quality was found for mean HR (94.9±21.8 bpm; r=0.034, p=0.758; F=0.413, p=0.663) or HRV (67.5±22.8 bpm; r=0.097, p=0.377; F=0.111, p=0.895). On per-segment analysis, sensitivity, specificity, positive predictive value (PPV), and negative predictive value (NPV) were 89.7% (26/29), 99.4% (355/357), 92.9% (26/28), and 99.2% (355/358), respectively, with excellent correlation (kappa=0.91) with ICA. Mean effective dose was 3.3±1.0 mSv.

**Conclusions:**

Prospectively ECG-triggered sequential dual-source CCTA provides diagnostic image quality and good diagnostic accuracy for detection of coronary stenosis in AF patients without significant influence by HR or HRV.

## Introduction

Atrial fibrillation (AF) is the most common form of cardiac arrhythmia and increases in incidence and prevalence with age [1, 2]. AF is usually associated with various kinds of structural heart diseases which can present with symptoms that mimic coronary artery disease (CAD). Therefore, demonstrating the presence or absence of CAD is of importance for AF patients, especially the elderly [2].

Coronary CT angiography (CCTA) as a noninvasive imaging examination has shown reliable diagnostic accuracy with regard to the detection and quantification of coronary artery lesions [3, 4]. In recent years, it has been widely investigated regarding its potential to exclude CAD in patients with a low or intermediate pre-test probability [5, 6]. However, insufficient temporal resolution at irregular heart rates has remained a main limitation of CCTA [7–11]. Furthermore, AF has previously been regarded as a contraindication for CCTA, due to increased heart rate variation (HRV) which can lead to severe motion artifacts [12–14]. Second-generation dual-source CT with two x-ray tubes and faster rotation times (280 ms) offers higher temporal resolution (75 ms), which is expected to decrease motion artifacts in patients with high HR as well as patients with AF.

Retrospective ECG-gated helical scan protocols remain the most common technique for CCTA in AF patients [15]. A recent meta-analysis showed that CCTA has high diagnostic accuracy in patients with AF, however, due to the retrospective nature of the current protocols, it is also associated with significantly higher effective radiation dose than in patients with sinus rhythm [15].

Some studies have demonstrated that prospectively ECG-triggered dual-source CCTA is feasible in patients with AF [16, 17]. However, the influence of HR and HRV on the image quality of prospectively ECG-triggered sequential dual-source CCTA in AF patients has not been assessed. Furthermore, the diagnostic performance of this scan protocol in patients with AF has not been evaluated.

Thus, the purpose of this study was to evaluate the effects of mean HR and HRV on image quality and diagnostic accuracy of prospectively ECG-triggered sequential dual-source CCTA in patients with AF.

## Materials and Methods

### Patients

The study protocol was approved by the ethics committee of Beijing Anzhen Hospital, and written informed consent was provided by all patients. Patients were excluded if they had a history of allergic reactions to iodine-containing contrast medium, an unstable clinical condition, renal insufficiency (serum creatinine >120 μmol/L), inability to hold breath (n = 3), previous stent implantation (n = 3) or bypass surgery, or were pregnant. A total of 85 patients with persistent AF were enrolled (49 women, 36 men; 62.1 years ± 9.5 [range, 34–83 years]). All patients underwent CCTA using second-generation dual-source CT. The mean body mass index (BMI) was 24.2 kg/m^2^ ± 3.9 (range: 17.3–36.3 kg/m^2^). Of these 85 patients, 42 underwent CCTA prior to valve surgery; 18 patients were evaluated for the treatment of AF; one patient underwent imaging for evaluation of hypertrophic cardiomyopathy; one patient was examined for surgical management of congenital heart disease; and 23 patients were examined due to suspected CAD. All of the patients have had AF for longer than 1 year. Thirty of these patients were scheduled to undergo additional conventional invasive coronary angiography (ICA) due to suspected CAD.

### Coronary CT angiography

All CT examinations were performed using a second-generation dual-source 128-slice CT scanner (Somatom Definition Flash, Siemens Healthcare, Forchheim, Germany). No drugs indicated for heart rate regulation were administered prior to the CT examination. The scan range was from the level of the tracheal bifurcation to the diaphragm. A 40–60 ml bolus of Iopromide (370 mg/ml; Bayer Schering Pharma, Berlin, Germany) injected into an antecubital vein at a flow rate of 3.5–5 ml/sec using a dual-head power injector (Stellant, Medrad, Inianola, USA) and followed by 30 ml of saline solution was continuously injected at the same flow rate.

The bolus-tracking technique was used. CT image acquisition initiated automatically after the signal attenuation reached the predefined threshold of 120 HU. The signal attenuation was measured by a region of interest in the ascending aorta. Data were acquired in craniocaudal direction with a detector collimation of 2 × 64 × 0.6 mm thickness and a gantry rotation time of 280 msec.

The tube voltage and tube current were modulated according to the patient’s body mass index (BMI). Patients with a BMI≥30 kg/m^2^ (10 cases) were scanned with a tube voltage of 120 kV and tube current 370–400 mAs, patients with a BMI<30 kg/m^2^ (64 cases) with 100 kV and 330–370 mAs, and patients with a BMI<20 kg/m^2^ (11 cases) with 80 kV and 330–350 mAs.

A prospectively ECG-triggered sequential scan with padding technique was performed. Imaging data was acquired using absolute phase acquisition (200 ms to 400 ms of the R-R interval). The ECG was automatically recorded during data acquisition. The highest HR, lowest HR, and average HR of cardiac cycles were noted. The HRV was calculated by subtracting the lowest HR from the highest HR. The CT examinations were performed in all patients without complications.

Images were reconstructed with a slice thickness of 0.75 mm, a reconstruction increment of 0.5 mm, a smooth convolution kernel (I26f), and a sinogram affirmed iterative reconstruction algorithm with a strength level of 2 (SAFIRE, Siemens Healthcare). The images were first reconstructed using automatic best systolic phase selection (BestPhase, Siemens Healthcare) and the manual multiple-phases reconstruction method was used for vessel segments with poor image quality (36 patients). The images were reviewed by a radiologist with more than 5 years of experience in cardiac imaging and the imaging data with the best image quality was transferred to a post-processing workstation (MMWP, Siemens Healthcare) for further analysis.

The CT dose index (CTDI) and dose-length product (DLP) were displayed by the CT system and the effective dose (ED) was calculated by means of a conversion factor of 0.014 mSv/(mGy·cm) [18].

### CCTA data analysis

For CCTA data analysis, 18 coronary segments were assessed according to the guidelines of the Society of Cardiovascular Computed Tomography (SCCT) [19], and all segments with a diameter of ≥1.5 mm at their origin were included. All reconstructed images were evaluated and classified by two independent readers, each with more than 5 years of experience in cardiac CT.

Original transverse images, multiplanar reformations, curved multiplanar reformations, and volume-rendered images were used for the evaluation of CCTA image quality and coronary artery lesions. Image quality for each coronary artery segment was qualitatively evaluated using a 4-point grading scale as follows [20]: A score of 1 corresponded to absence of motion artifacts or noise-related blurring, excellent vessel opacification, and no structural discontinuity; a score of 2, minor motion artifacts or noise-related blurring, good vessel opacification, and minimal vessel discontinuity; a score of 3, some motion artifacts or noise-related blurring, fair vessel opacification or moderate structural discontinuity, but sufficient delineation of the individual segments; and a score of 4 was given to segments that could not be evaluated (non-diagnostic) because of a lack of vessel wall definition due to marked motion artifact, poor vessel opacification, prominent structural discontinuity, and severe image noise-related blurring. The segments with a score of 4 were not included in further diagnostic analysis.

The CCTA images were also evaluated for the presence of significant stenosis. Similar to invasive coronary angiography (ICA), significant stenosis was defined as a narrowing of the coronary artery lumen >50%. Each vessel was evaluated on at least two planes (one parallel and one perpendicular to the course of the coronary vessel).

In case of any disagreement in data analysis (image quality and lumen stenosis), datasets were reviewed by the two readers to reach a consensus.

### Conventional invasive coronary angiography

A total of 30 patients underwent ICA 3–7 days after their CT examination. The decision for ICA was made by the treating physician in accordance with the patient independent from CCTA results. The ICA was performed using standard methods by a radiologist with more than 5 years of experience in the interpretation of coronary angiograms and who was blinded to the results of the CCTA. The coronary arteries were also segmented according to the guidelines of the SCCT [19] as the standard of reference to CCTA. The vessel segments were evaluated using the quantitative coronary analysis method [21]. The diameter stenosis was determined by taking into account the vessel luminal narrowing from two different orthogonal projections. Coronary artery lesions causing a reduction in lumen diameter over 50% were considered to be significant stenosis.

### Intermodality comparisons

The diagnostic ability of CCTA was compared with results of ICA according to three levels: (1) on a per-segment level, comparing each segment in every vessel; (2) on a per-vessel level, evaluating the presence of significant lesions in each of the major coronary vessels; and (3) on a per-patient level, examining the presence of any significant lesion in a given patient.

### Statistical analysis

Statistical analysis was performed with commercially available statistical software (SPSS, version 16.0 for Windows; SPSS Inc, Chicago, IL, USA). Quantitative variables were expressed as means ± standard deviations or median ± interquartile range; and categorical variables were expressed as frequencies or percentages. A p-value less than 0.05 indicated a statistically significant difference.

For each patient and each coronary branch, Pearson (normally distributed data) or Spearman (non-normally distributed data) correlation analysis was performed to compare the image quality score with the mean HR and HRV during CCTA. The distribution of data were determined by using the Kolmogorov-Smirnov test. The effect of mean heart rate and heart rate variation on image quality in each patient was evaluated by using the UNIANOVA analysis.

Sensitivity, specificity, positive predictive value (PPV), and negative predictive value (NPV) of CCTA were calculated. McNemar’s test was used to evaluate a significant difference between dual-source CCTA and ICA for detection of coronary artery stenosis. Interobserver agreement in subjective image quality grading and intermodality agreement between CCTA and ICA was determined by calculating κ statistics. According to Landis and Koch [22], κ = 0 indicates poor agreement, κ = 0.01–0.20 indicates slight agreement, κ = 0.21–0.40 indicates fair agreement, κ = 0.41–0.60 indicates moderate agreement, κ = 0.61–0.80 indicates good agreement, and κ = 0.81–1.00 indicates excellent agreement.

## Results

### Patient characteristics

All patients showed rapid and irregular heart rates. The mean HR was 94.9 bpm ± 21.8 (range, 51–141 bpm) and the mean HRV was 67.5 bpm ± 22.8 (range, 6–119 bpm). In 53 patients (62.3%), 250–300 msec after R wave was identified as the best reconstruction interval with the fewest artifacts. In 16 patients (18.8%), the best reconstruction phases were less than 250 msec after R wave and >300 msec in the remaining 16 patients. The mean number of scan slabs was 4 (range, 3–5 slabs) and the average patient effective dose was 3.3 mSv ± 1.0 (range, 0.9–8.9 mSv). Patient characteristics are summarized in Table 1.

**Table 1:** Patient characteristics.

	Data of patients
**Age (year)**	62.1± 9.5 (34–83)
**Female/male** *	49/36
**BMI(kg/m** ^**2**^ **)**	24.2±3.9 (17.3–36.3)
**Mean Heart rate (bpm)**	94.9±21.8 (51–141)
**Heart rate variation (bpm)**	67.5± 22.8 (6–119)
**Scan heart beat** ^Δ^	4±1 (3–5)
**CTDIvol (mGy)** ^Δ^	16.91±6.1 (4.9–46.0)
**DLP(mGy** * **cm)** ^Δ^	233.5±71.8 (67.0–635.0)
**Effective dose (mSv)** ^Δ^	3.3±1.0 (0.9–8.9)

Data are means± standard deviations.

* Data is numbers of patients.

^**Δ**^ Data are median± interquartile range.

### Image quality of CCTA

In the 85 patients enrolled, a total of 1102 segments with a diameter of at least 1.5 mm were evaluated (90 segments were identified with a diameter of less than 1.5 mm). Of these 1102 segments, image quality was considered excellent (a score of 1) in 775 (70.3%) segments, good in 274 (24.9%), moderate in 45 (4.1%), and poor in 8 (0.7%, including 2 RCA segments, 2 LAD artery segments, and 4 LCX artery segments). The 8 segments with poor image quality belonged to 8 separate patients with different HR and HRV. The image quality score of coronary artery segments in different HR and HRV is summarized in Table 2. The segments with poor image quality were not considered for further diagnostic analysis. There was a good agreement for image quality scoring between the two reviewers (Kappa = 0.72).

**Table 2:** Image Quality Scores of Dual Source CCTA and the effect of Mean Heart Rate and Heart Rate Variation on image quality.

Image Quality Score	Total (%) (n = 85)	Mean heart rate (%)			Heart rate variation (%)		
		<80bpm (n = 18)	80-100bpm (n = 40)	>100bpm (n = 27)	<50bpm (n = 17)	50-70bpm (n = 30)	>70bpm (n = 38)
**1**	70.3	70.0	73.6	65.5	69.4	72.5	69.1
	(775/1102)	(159/227)	(388/527)	(228/348)	(154/222)	(277/382)	(344/498)
**2**	24.9	23.8	21.1	31.3	24.3	23.0	26.5
	(274/1102)	(54/227)	(111/527)	(109/348)	(54/222)	(88/382)	(132/498)
**3**	4.1	4.9	4.6	2.9	5.4	3.4	4.0
	(45/1102)	(11/227)	(24/527)	(10/348)	(12/222)	(13/382)	(20/498)
**4**	0.7	1.3	0.7	0.3	0.9	1.1	0.4
	(8/1102)	(3/227)	(4/527)	(1/348)	(2/222)	(4/382)	(2/498)
**Per-patient Analysis**	Mean Score	1.38±0.22	1.32±0.31	1.39±0.26	1.38±0.30	1.33±0.24	1.36±0.29
	F value	0.413			0.111		
	p-value	0.663			0.895		

In our study, motion artifacts were responsible for the resulting poor image quality. The distal segments of the LCX (4 segments) were the most influenced by motion artifacts. In addition, stair-step artifacts also caused a deterioration of image quality. Ninety-four vessel segments were evaluated as good image quality instead of excellent due to stair-step artifacts. The LM coronary artery and the proximal segments of each coronary artery branch were the least affected sections with the best image quality.

### Relationship between image quality and heart rate

There was no significant difference in the mean image quality score per patient during different mean HR and HRV groups (P>0.05) (Table 2).

**Fig 1:**
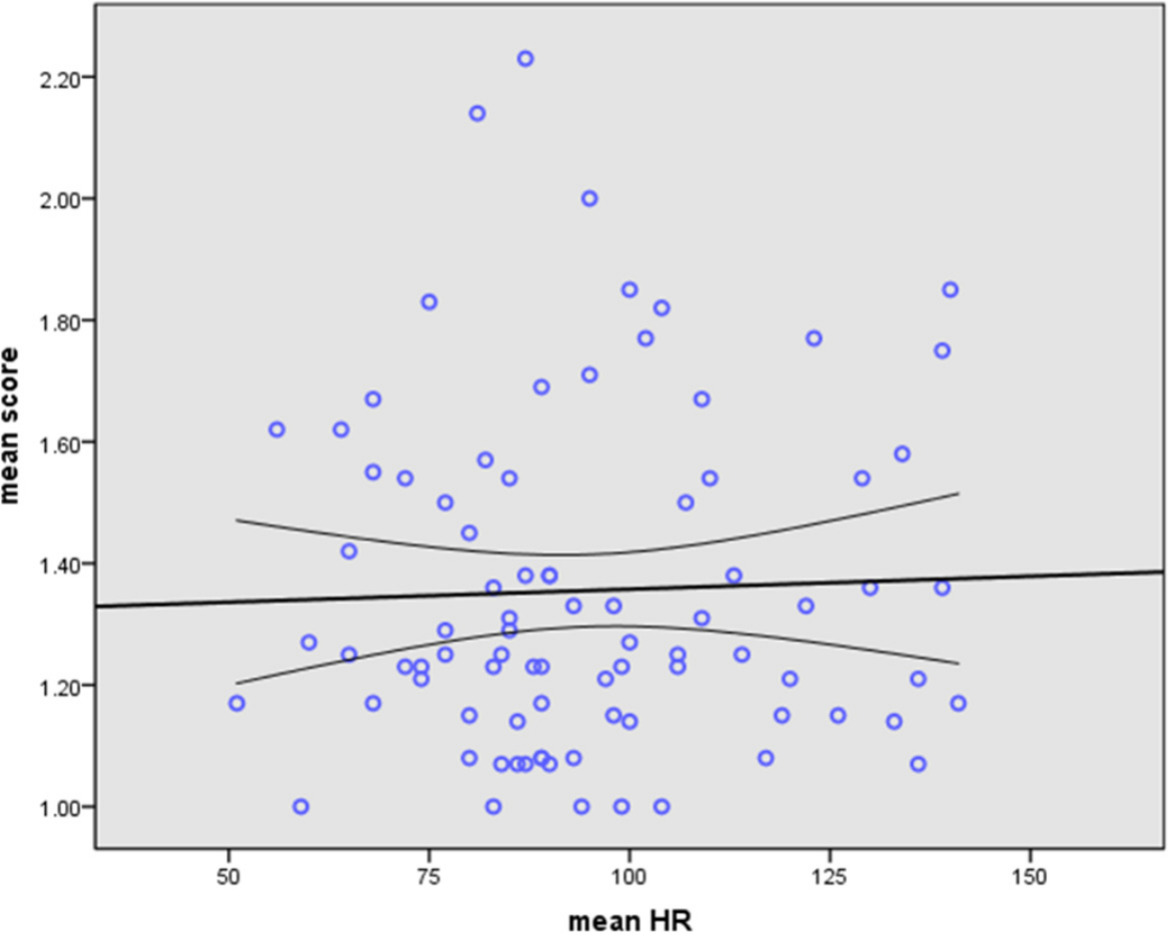
The Linear regression analysis of image quality versus HR. No significant linear correlation between image quality and mean HR in AF patients by using prospectively ECG-triggered dual-source CT (Pearson correlation, r = 0.034, P = 0.758).

**Fig 2:**
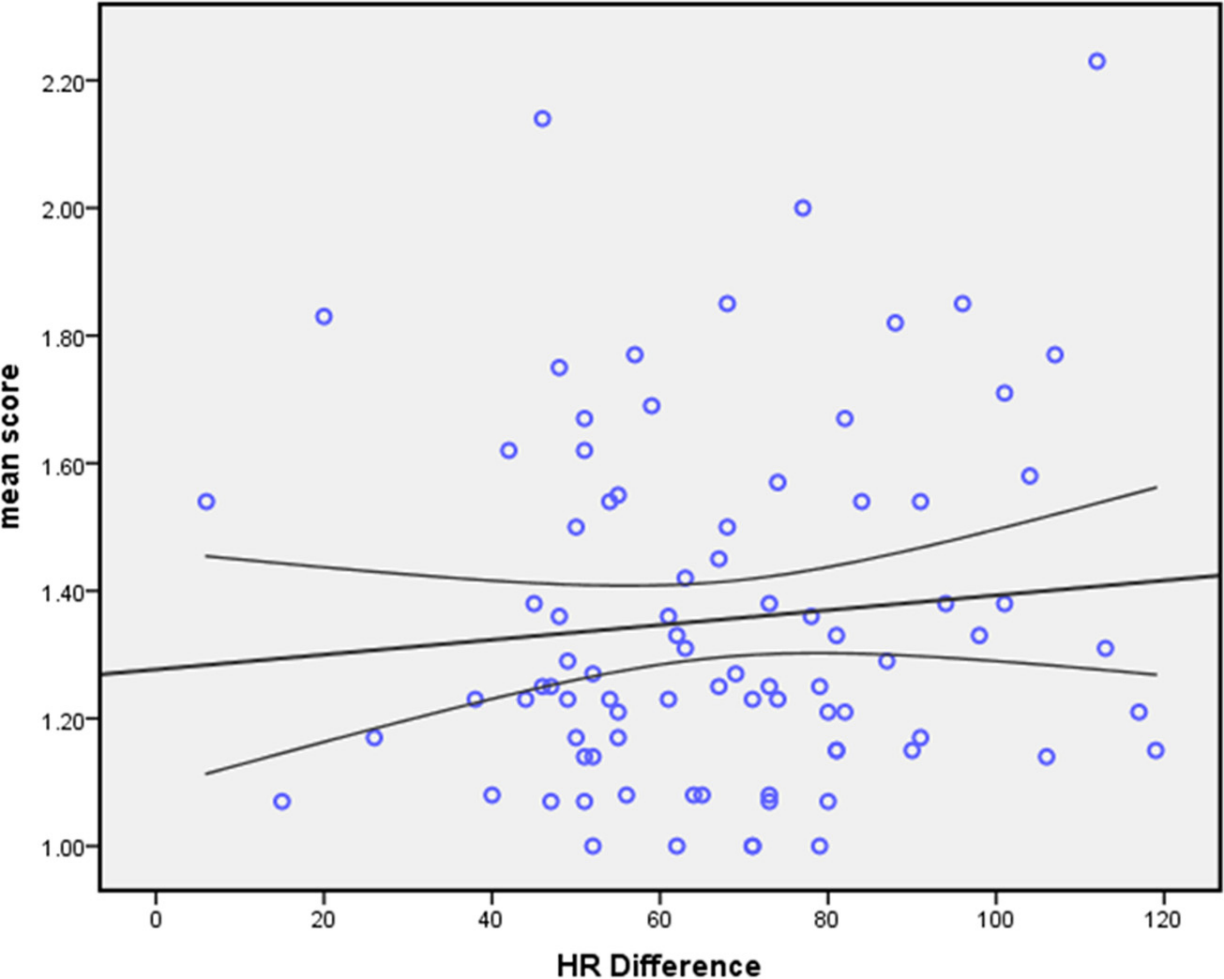
The Linear regression analysis of image quality versus HRV. No significant linear correlation between image quality and HRV in AF patients using prospectively ECG-triggered dual-source CT. (Pearson correlation, r = 0.097, P = 0.377).

No significant correlation was observed between mean HR and mean image quality scores for all coronary segments in each patient (P>0.05) (Fig 1) or for each branch of coronary artery (P>0.05) (Table 3). Similarly, there was no significant correlation between HRV during CCTA and mean image quality scores for all coronary segments (P>0.05) and branches (Fig 2).

**Table 3:** Correlation between image quality of Dual Source CCTA with Mean Heart Rate and Heart Rate Variation.

Correlation Analysis		Mean heart rate		Heart rate variation	
		r value	p-value	r value	p-value
**Segment**	Total	0.034*	0.758	0.097*	0.377
**Vessel**	RCA	0.071^Δ^	0.520	0.033^Δ^	0.762
	LAD	-0.014*	0.902	0.068*	0.539
	LCX	0.065^Δ^	0.556	0.158^Δ^	0.148

*Pearson correlation result

^**Δ**^spearman correlation result.

### Diagnostic performance of dual-source CCTA

No significant stenosis was found in 20 patients. Fifty-nine vessel segments were considered to have ≤50% lumen narrowing. Twenty-nine segments were found to have a significant diameter reduction of more than 50%. No vessel segment with total occlusion was observed.

**Fig 3:**
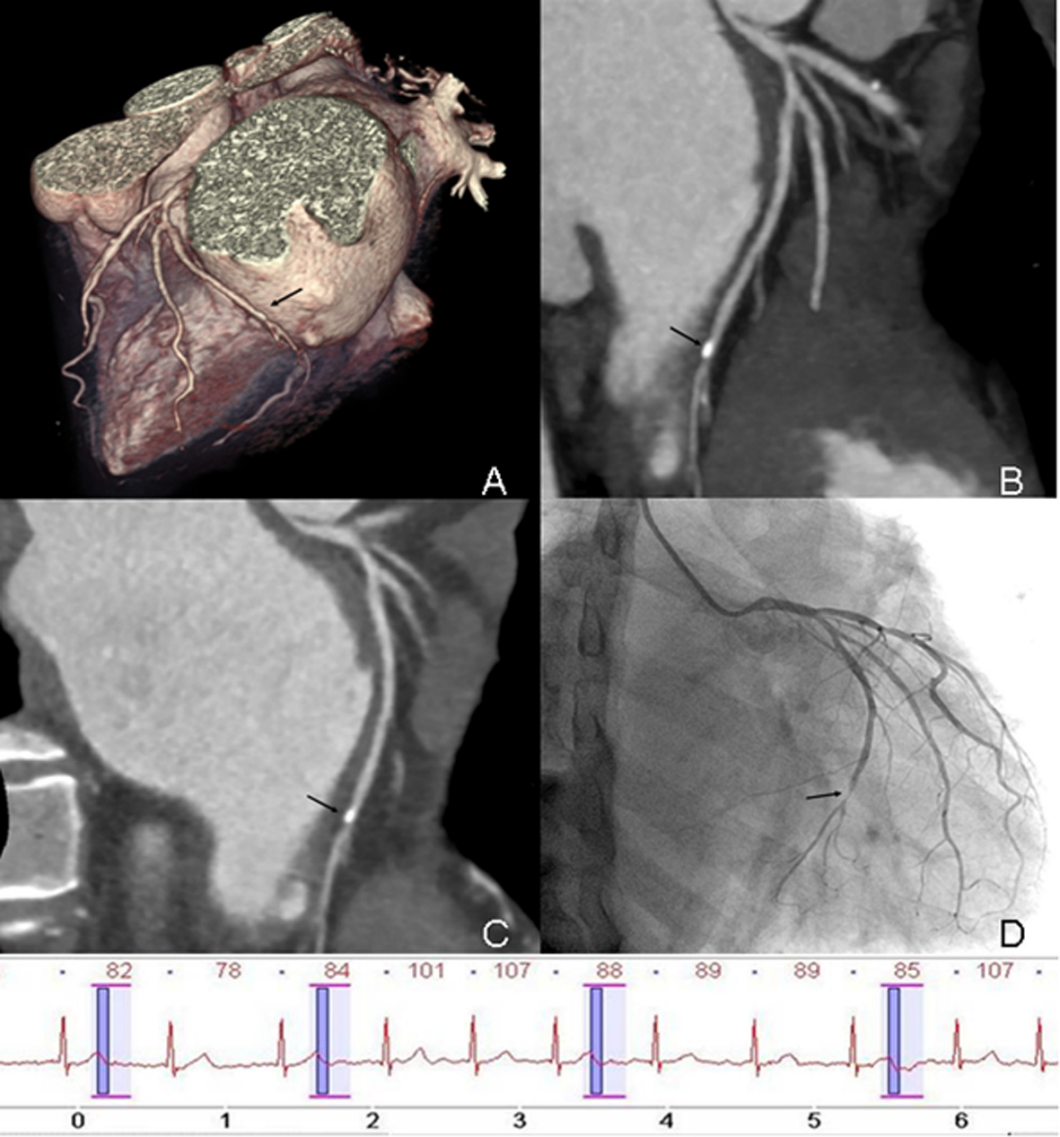
Prospectively ECG-triggered dual-source CCTA of a 60-year-old man with AF. Mean HR was 86 bpm (range, 60–107 bpm). Images reconstructed at 240 ms after R wave. Volume-rendered (A), maximum intensity projection (B) and curved multiplanar reformation (C) image of LCX (black arrow) show the vessel lumen was overlapped by calcified plaque in the distal segment. Conventional coronary angiogram (D) shows significant stenosis (>50%) in distal segment of LCX (black arrow). The ECG information was recorded during data acquisition.

**Fig 4:**
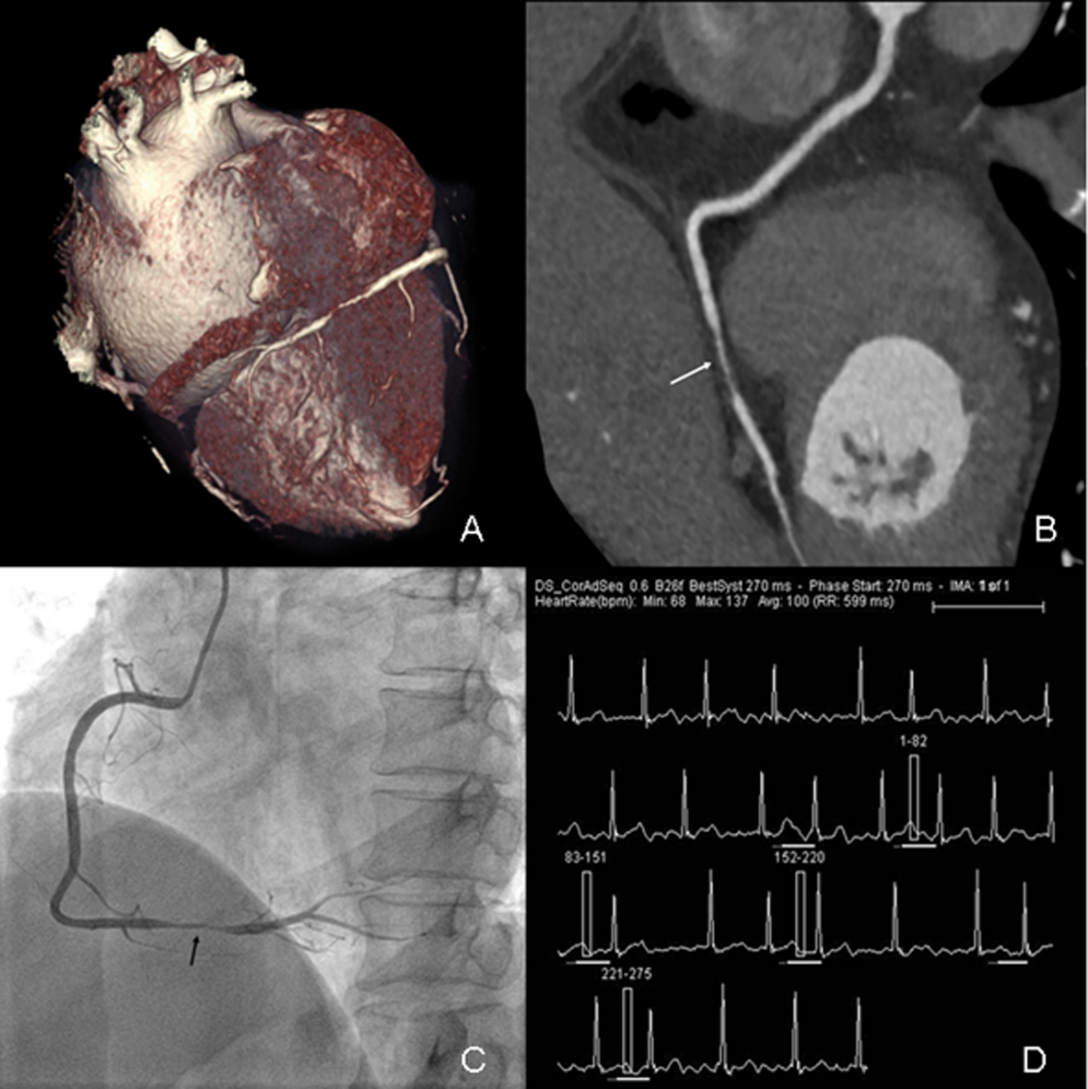
Prospectively ECG-triggered dual-source CCTA of a 57-year-old woman with AF. Mean HR was 100 bpm (range, 68–137 bpm). Images reconstructed at 270 msec after R wave. Volume-rendered (A) and curved multiplanar reformation (B) images of RCA (white arrow) show atherosclerosis lesion (stenosis>50%) in distal segment. Conventional coronary angiogram (C) shows significant stenosis (>50%) in distal segment of RCA (black arrow). The ECG information (D) was recorded during data acquisition.

The sensitivity, specificity, PPV, and NPV values of CCTA are shown in Table 4. On a per-segment basis, three vessel segments with poor image quality on CCTA were ruled out due to the calculation of diagnostic accuracy. The sensitivity, specificity, PPV, and NPV for CCTA were 89.7%, 99.4%, 92.9%, and 99.2%, respectively. On a per-vessel basis, they were 83.3%, 97.0%, 90.9%, 94.1%, respectively, and on a per-patient basis, they were 81.8%, 94.7%, 90.0%, and 90.0%, respectively. There was good to excellent intermodality agreement between CCTA and ICA in terms of detection of significant stenosis (κ = 0.91, 0.83, 0.78) (Table 4, Figs 3 and 4).

**Table 4:** Performance of Prospectively ECG-triggered Dual Source CCTA for Diagnosing CAD in Patients with AF.

Analysis	Result						Parameter				
	True- positive	True- negative	False- positive	False- negative	Non- evaluable (positive)	Non- evaluable (negative)	Sensitivity	Specificity	PPV	NPV	κ
							(%)	(%)	(%)	(%)	
							[95%CI]	[95%CI]	[95%CI]	[95%CI]	
Per-segment (n = 386)	26	355	2	3	1	2	89.7	99.4	92.9	99.2	0.91
							(26/29)	(355/357)	(26/28)	(355/358)	
							[78.7–100]	[98.4–100]	[83.4–100]	[98.3–100]	
Per-vessel (n = 90)	20	63	2	3	1	1	83.3	97.0	90.9	94.1	0.83
							(20/24)	(64/66)	(20/22)	(64/68)	
							[68.4–98.2]	[92.9–100]	[79.1–100]	[88.5–99.7]	
Per-patient (n = 30)	9	17	1	1	1	1	81.8	94.7	90.0	90.0	0.78
							(9/11)	(18/19)	(9/10)	(18/20)	
							[59.0–100]	[84.7–100]	[71.4–100]	[76.9–100]	

## Discussion

In our study, we observed that prospectively ECG-triggered sequential second-generation dual-source CCTA reliably provides diagnostic image quality in AF patients without significant influence by mean HR and HRV. In addition, we found good to excellent intermodality diagnostic agreement between CCTA and ICA for the detection of coronary artery lesions. Our results imply that prospectively ECG-gated sequential second-generation dual-source CCTA is a dependable technique to rule out coronary artery stenosis in patients with AF independent of HR and HRV.

Throughout the last decade, retrospectively ECG-gated CCTA with low pitch and a wide exposure window has been regarded as a more reliable choice for CCTA in AF patients in order to minimize the risk of motion artifacts. However, the higher radiation dose of this scan protocol has raised serious concerns [23]. In recent years, prospectively ECG-triggered scan protocols with their lower radiation dose have become more popular for CCTA [24, 25].

With the advantage of the higher temporal resolution and wider detector of the second generation dual-source CT, some studies [16, 26] have demonstrated that prospectively ECG-triggered sequential scanning with the padding technique is feasible in patients with AF. In our study, 99.3% of the coronary vessel segments were of diagnostic image quality, and poor image quality was found in only 8 patients with different HR and HRV. This result is consistent with findings of prior studies [16]. Furthermore, the results of our study indicate that when using prospectively ECG-triggered dual-source CCTA in AF patients, image quality does not significantly linearly correlate with mean HR or HRV. In addition, there was no significant difference in mean image quality score per patient in different HR and HRV groups. These results indicate that mean HR and HRV do not have a significant effect on the image quality of CCTA in AF patients.

Diagnostic image quality in patients with AF may be obtained throughout a wide range of mean HR and HRV. This finding may be related to the application of the adaptive prospectively ECG-triggered sequential scanning method. This method is part of a newer generation of prospectively ECG-triggered techniques and has been developed to allow adaption to cardiac rhythm irregularity. Compared with the conventional prospectively sequential scan mode, an extended exposure window allows for more flexibility in choosing different cardiac phases for image reconstruction. Arrhythmia compensation was provided by automatically omitting or repeating the scans on demand. The use of monosegment reconstruction protocol, which does not merge data from adjacent heartbeats in different cardiac phases, also improves image quality with irregular heart rates.

Although higher temporal resolution and increased detector width were achieved with prospectively ECG-triggered dual-source CT technology, vessel segment discontinuity and residual heart motion artifacts could not be abolished completely. There are several factors related to impairments in image quality. First, multiple scan slabs during the prospectively sequential scan cause vessel discontinuity. In our study, only 3–5 scan slabs were included during data acquisition, but the ‘‘stair-step” artifacts could not be completely eliminated. The quality of 94 segments were not evaluated as having excellent image quality due to “stair-step” artifacts. Second, in our study, 4 distal segments of LCX were evaluated with poor image quality. It may have been caused by the smaller size of the distal segment of LCX, short motion-free period [27, 28], and/or close proximity to the enlarged left atria [29–30]. Third, the phase and width of the exposure window are closely related to image quality. Inappropriate selection of the cardiac phase can be a potential source of image-degrading artifacts. In patients with higher heart rates, the temporal variation during end-systole in each heartbeat is less than in mid-diastole. The exposure window should be set at end-systolic phase in order to get superior image quality for coronary artery imaging [31]. A wider exposure window provides the feasibility of multiphase reconstruction, but also results in a higher radiation dose. Therefore, it is beneficial to use the adequately narrowed exposure windows based on HR [32]. In many studies, the images with best image quality were obtained at end-systolic phases in AF patients [14, 16, 33, 34]. Consistent with these studies, end-systolic phase acquisition (200–400 ms in R-R interval) was used in our study and the images with highest quality were most commonly obtained from 250 ms to 300 ms of the R-R interval (62.3% patients).

Technical innovations in CT equipment have improved spatial and temporal resolution while reducing radiation exposure [35, 36]. There are many factors influencing the x-ray radiation dose including the tube voltage, tube current, scan range, ECG gating, slice thickness, overlap and pitch. In our study, besides the use of the prospectively ECG-triggered scan protocol, we modulated the tube voltage and current based on BMI and set the number of scan slabs as low as possible. In our study, the mean effective dose was 3.3 mSv, which is comparable to the mean effective dose in patients with sinus rhythm examined with the same protocol [37].

CCTA is regarded as a reliable modality for non-invasive evaluation of CAD, especially in patients with a low to intermediate pre-test likelihood (15). In these patients, CCTA can rule out substantial stenosis and may help to omit ICA [38]. In our study, nearly 50% of patients (42 cases) who suffered from valvular disease opted for CCTA instead of the ICA to rule out CAD prior to valvular surgery.

In our study, the diagnostic accuracy of prospectively ECG-triggered dual-source CCTA in patients with AF is similar to that of retrospectively ECG-gated CCTA [15]. Besides severe motion artifacts, calcification is another main factor that may impair image quality and image interpretation. In our study, although calcified lesions were rare, the presence of calcification did render image interpretation more difficult, with overestimation of stenosis in 2 segments and underestimation of 2 segments despite motion-artifact-free image quality (Fig 3).

There are some limitations to this study which should be mentioned. First, the number of included patients for the diagnostic analysis was relatively small and had a low prevalence of CAD. This may have influenced especially the sensitivity and specificity results of CCTA for the detection of significant stenosis. It will be useful to verify our results in other studies including larger patient populations. Second, the subjective image quality scoring system may potentially bias the final result. Furthermore, motion artifacts may appear like normal tissue and may therefore have been under-reported; we evaluated multiple reconstruction phases to mitigate this limitation. Finally, our results are only applicable to the second-generation dual-source CT used in our study and results may vary with other systems.

## Conclusion

Prospectively ECG-triggered sequential CCTA provides diagnostic image quality in AF patients without significant influence by HR or HRV, shows a good diagnostic accuracy in comparison with ICA, and may therefore be a beneficial technique to rule out CAD at a relatively low radiation dose in AF patients prior to cardiac surgery.
